# Polylactide/Montmorillonite Hybrid Latex as a Barrier Coating for Paper Applications

**DOI:** 10.3390/polym8030075

**Published:** 2016-03-04

**Authors:** Davide Bandera, Veronika R. Meyer, David Prevost, Tanja Zimmermann, Luciano F. Boesel

**Affiliations:** 1 Empa, Swiss Federal Laboratories for Materials Science and Technology, Laboratory for Applied Wood Materials, Überlandstrassse 129, CH-8600 Dübendorf, Switzerland; dav.bande@gmail.com; 2 Empa, Swiss Federal Laboratories for Materials Science and Technology, Laboratory for Biointerfaces, Lerchenfeldstrasse 5, CH-9014 St. Gallen, Switzerland; 3 Empa, Swiss Federal Laboratories for Materials Science and Technology, Laboratory for Protection and Physiology, Lerchenfeldstrasse 5, CH-9014 St. Gallen, Switzerland; veronika.meyer@empa.ch; 4 Cham Paper Group Schweiz AG, Fabrikstrasse, CH-6330 Cham, Switzerland; prevost.david@cham-group.com

**Keywords:** hybrid latex, paper coating, polylactide/montmorillonite hybrid coating, water-based coating latex, food packaging, water vapor transmission rate

## Abstract

We developed a paper coating for the potential application in food packaging based on polylactide and montmorillonite. It is applied to the paper in the form of a stable, water-based latex with a solid content of 25–28 wt %. The latex is prepared from a commercially available polylactide, surfactants, montmorillonite, a plasticizer, chloroform (to be removed later) and water by an emulsion/solvent evaporation procedure. This coating formulation is applied to the paper substrate by bar-coating, followed by hot-pressing at 150 °C. The coated papers achieved up to an 85% improvement in water vapor transmission rates when compared to the pristine papers. The coating latex is prepared from inexpensive materials and can be used for a solvent-free coating process. In addition, the ingredients of the latex are non-toxic; thus, the coated papers can be safely used for food packaging.

## 1. Introduction

The contamination of food by unexpected or even toxic compounds migrating not from the environment, but from the packaging is a highly unwanted phenomenon; nevertheless, it is observed by food control laboratories. Papers used for the packaging of food are usually coated; the coatings are needed as a barrier against the permeation (from both sides) of water vapor and/or oxygen, thus preserving the quality and preventing the degradation of packaged food. Current solutions for improving the barrier properties include the use of composite materials, but most solutions on the market are based on oil-derived polymer formulations. Such coatings can release phthalates, epoxides, styrene or mineral oil components [1,2,3,4,5]. It is therefore necessary to develop innovative coatings for paper products based on biopolymers [6], e.g., formulations based on starch, gelatin or wheat gluten [7]. The consumer goods market with barrier papers for direct food contact applications is rated as an important future market with strong growth.

Our goal was to develop an inexpensive and eco-friendly water-based dispersion (latex) composed of a biopolymer and a clay material that can be used for the coating of paper. Previous experiments had shown that layered silicates have interesting properties [8,9,10,11,12,13,14,15]. The inspirational sources were natural inorganic-organic composites, like nacre [9], which is found in mother of pearl and other seashells. Their hierarchical structure imparts exceptional mechanical and barrier properties. These constructs resemble that of an array of bricks glued together by biopolymers. In such a system, gases would have to pass a long and tortuous pathway through the structure. Mimicking similar constructions, as has been shown previously for nanofibrillated cellulose/clay systems [15,16], would allow for the preparation of materials with good barrier properties. However, the dispersion needs to be easily applicable to paper by one of the well-established techniques used in the paper industry, such as curtain coating, blade coating or rod coating. Each of these techniques requires specific operational parameters, like viscosity, solid content, *etc.*, which must be fulfilled by a newly-developed formulation. Our previous work on solvent-based methods allowed us to increase the water vapor barrier by a factor of ten [8]. These systems, however, involved coating from solutions, which, in the case of high barrier hydrophobic polymers, required the use of organic and halogenated solvents. These solvents, if present during the coating process, could migrate into the packaging and later into the food, posing health risks. Moreover, they are usually undesired solvents in the paper and packaging industry. Regarding composites with high oxygen barrier properties, these may be produced from water-based solutions or dispersions [12,14], therefore raising less concerns on environmental and health aspects.

To address the issues with high water vapor barrier coatings, we investigated the preparation of hybrid latex systems that could be applied as a coating for paper packaging. The investigated techniques for the preparation of a latex were the emulsion/solvent evaporation and the emulsion/precipitation procedures, of which the first was successful. We studied different mixtures of the following materials: various polylactides (PLA), various poly(3-hydroxybutyrate)-derived polymers and microfibrillated or microcrystalline cellulose as the biopolymer component; acetone or chloroform as the solvent; various surfactants admitted in the food industry; tributyl citrate as a plasticizer for the paper coating process; and pristine or organically-modified montmorillonite (MMT) as the clay component of the water-based latex. The relative amounts of the various latex ingredients were varied. Most combinations did not yield a stable latex or led to aggregation of the material. Here, we present the preparation and technical properties of the most promising formulation, namely a latex composed of PLA and pristine MMT, complemented with tributyl citrate as a plasticizing agent for the paper coating process.

## 2. Experimental Section

### 2.1. Chemicals and Instruments

The following chemicals were used for the preparation of the PLA/MMT latex:

Polylactide: NW PLA 4042 D, a poly-l-lactide containing about 1.4% d-lactic acid units and with a molar mass (*M*_w_) of 113,000 g/mol (NatureWorks, Minnetonka, MN, USA). Solvent: chloroform, stabilized with amylene, HPLC grade (Fisher Scientific, Loughborough, UK). Plasticizer: tributyl citrate (Merck, Zug, Switzerland). Surfactants: dioctyl sulfosuccinate sodium salt, ≥97% (Sigma-Aldrich, Buchs, Switzerland), and Igepal CO-630, which is branched nonylphenoxy poly(ethyleneoxy)ethanol (Sigma-Aldrich, Buchs, Switzerland). Montmorillonite: Dellite LVF, a pristine sodium MMT, and Dellite 43 B, an organically-modified MMT (OMMT), were supplied by Laviosa Chimica Mineraria, Livorno, Italy.

For the homogenization of the PLA/MMT dispersions, a SilentCrusher M was used (Heidolph Instruments, Schwabach, Germany).

For the application of the PLA/MMT latexes on glass surfaces (for the first tests of their barrier properties), a bar-coater, Coatmaster 509 MC, was used (Erichsen Testing Equipment, Hemer, Germany).

For the coating of the paper, a bar-coater, K 202 Control Coater, was used (RK PrintCoat Instruments Ltd, Royston, UK).

Paper qualities for coating: Kraft Lux (KL) or Silico NQ (SiNQ).

The following instruments were used for the characterization of the new products:

Scanning electron microscopy (SEM): Prior to observation, all samples were sputter coated directly with a platinum layer of about 5 nm (BAL-TEC MED 020 Modular High Vacuum Coating Systems, BAL-TEC AG, Liechtenstein) in Ar as a carrier gas at 5 × 10^−2^ mbar. SEM images were finally recorded with a FEI Nova NanoSEM 230 instrument (FEI, Hillsboro, OR, USA) at an accelerating voltage of 5 kV.

Energy-dispersive X-ray spectroscopy (EDX): Hitachi S-4800 (Hitachi GmbH, Rotkreuz, Switzerland), using an acceleration voltage of 15 kV. Before observation, specimens were coated with a thin (~5 nm) gold/palladium layer in a Leica ACE600 high vacuum coater (Leica GmbH, Vienna, Austria).

X-ray diffraction (XRD): PW 1729/1820 using 1.54 Å Cu Kα radiation (Philips, Eindhoven, The Netherlands).

Differential scanning calorimetry (DSC): DSC822^e^ (Mettler Toledo, Greifensee, Switzerland). The following three-step program was applied to all specimens: first heating from 0–210 °C at 10 °C /min; cooling to 0 °C at a rate of 10 °C /min; second heating to 210 °C at 10 °C /min. The glass- transition (*T*_g_), melting (*T*_m_) and crystallization (*T*_c_) temperatures were obtained from the first and the second heating cycles. The degree of crystallinity (*Χ*_c_) was calculated from the enthalpy of fusion of the first heating cycle as described in [8], using an enthalpy of fusion of a 100% crystalline PLA (94 J/g) [8].

Water vapor transmission rates (WVTR): The neat PLA/MMT films were investigated with a VTI-SA+ dynamic vapor sorption analyzer (TA-Instruments, New Castle, UK). Silica gel was placed in a small cup with a 10-mm inner diameter, 15-mm outer diameter and 10-mm height, which was then sealed by a piece of PLA/MMT film attached to the cup using double-sided adhesive tape. The conditions in the chamber were 85% RH and 23 °C. Each sample was investigated twice. The WVTR of coated paper was measured according to the ASTM Standard E-96 using the “desiccant method”. The area of the films (at least two per sample) was adjusted by using aluminum masks with the desired area. Tests were carried out at an external relative humidity of 50% and temperature of 23 °C. In either case, by measuring the mass gain of the silica gel over time under a controlled atmosphere, the WVTR could be calculated from the slope of a straight line obtained by linear regression of mass gain *versus* time and then by dividing the slope by the exposed area of the films.

Viscosity: Physica MCR 301 (Anton Paar, Graz, Austria), an instrument for oscillatory rheometry with parallel plates (25 mm), was used to determine the viscosity of the latexes. A frequency sweep program was used with the following conditions: shear rate 50 s^−1^, temperature 23 °C and angular frequency between 0.05 and 500 rad/s.

Gel permeation chromatography (GPC): A Viscotek VE 2001 instrument with refractive index detection was used (Viscotek, Weingarten, Germany). Chromatography was performed with 3 coupled polystyrene columns, all of an 8-mm i.d. and 30-cm length, a 10-μm particle diameter, of pore diameters of 10^3^, 10^5^ and 10^7^ Å, respectively (PSS Polymer Standards Service, Mainz, Germany). The eluent was 1 mL/min chloroform, temperature 35 °C.

### 2.2. Preparation of the PLA/MMT Latex

The emulsion/solvent evaporation procedure turned out to be the optimum method for the preparation of the hybrid latex. We describe the preparation of the most promising latex composition.

The first step consisted of the preparation of a PLA/plasticizer/surfactant solution. The PLA was dried under vacuum at 80 °C overnight. Due to the relatively high crystallinity of the material (21%–29%, as determined by DSC), it was necessary to use chloroform as the solvent, which allowed preparing solutions with a PLA content of up to 20% *m*/*v*. Six grams of dried NW PLA 4042 D were stirred overnight in 30 mL of chloroform at 45 °C for complete dissolution. Three hundred sixty mg of tributyl citrate (plasticizer), 179 mg of dioctyl sulfosuccinate sodium salt and 31 mg of Igepal CO-630 (surfactants) were dissolved in 2–3 mL of chloroform by magnetic stirring. The PLA and additive solutions were mixed and stirred for *ca*. 5 min at 45 °C until a homogeneous solution was obtained.

In the second step, the hybrid PLA/MMT latex was prepared. Thirty milliliters mL of MMT/water dispersion (0.78% *m*/*v*) were added to the chloroform solution. The resulting biphasic mixture was homogenized at room temperature at 12,000 rpm for 2–3 min. The dispersion was transferred to a beaker and stirred at 45 °C until the chloroform was evaporated. This process yielded an aqueous latex composed of 20% PLA, 1.2% plasticizer, 0.7% surfactants and 0.8% MMT. In other words, the solid content of the latex consisted of 88.2% PLA, 5.3% plasticizer, 3.1% surfactants and 3.4% MMT (all values are wt %).

### 2.3. Coating Procedures

For the preparation of self-supporting PLA and PLA/MMT films, the various latexes were bar-coated on glass surfaces at a drawing speed of 15 mm/s and a temperature of 70 °C. After the coating process, the films were dried in an oven at 150 °C for 5 min. Finally, they were removed from the glass for the investigation of their WVTR properties.

The most promising latexes were then coated on paper in the K 202 Control Coater at Cham Paper Group. A grooved bar (GH-Beschichtungstechnik GmbH, Legau, Germany) was used, with the coating speed set at 33 mm/s. The papers were coated with 6 layers; after each step, the coating was dried in an infrared oven (180 °C, 30 s). The 6-layer coated papers were then annealed in a hot press at 150 °C for 5 min.

## 3. Results and Discussion

### 3.1. Properties of the PLA/MMT Latex

**Figure 1:**
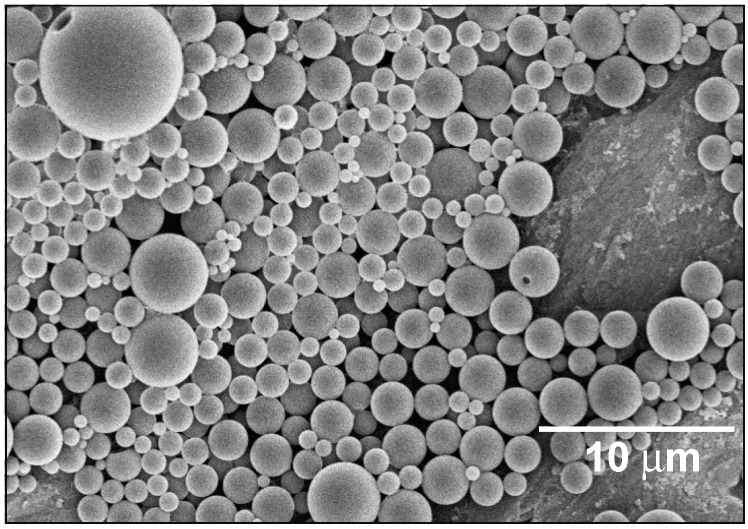
SEM image of a PLA/MMT hybrid latex.

**Figure 2:**
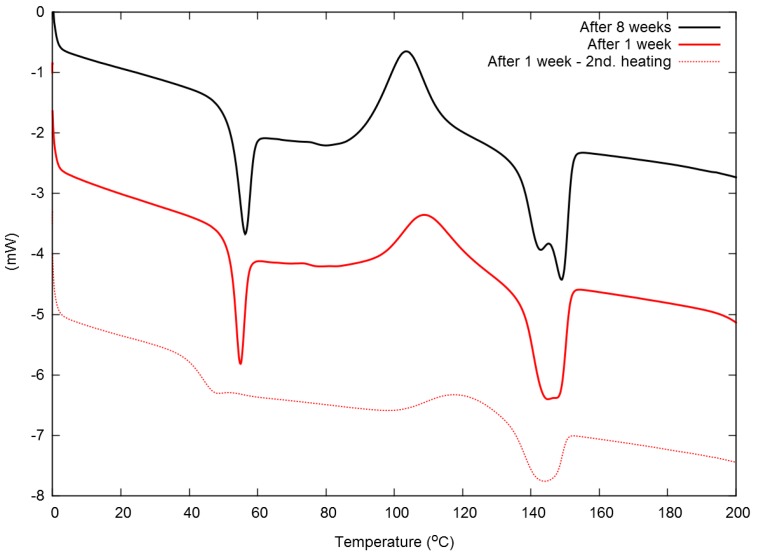
DSC curves (1st and 2nd heating) of PLA/MMT films after one and eight weeks of latex ageing. The curves were displaced vertically for easier reading.

The particles of this hybrid latex had a size between 0.5 and 6 μm; see Figure 1. The latex is very stable, and although it tends to stratify over time, it can be re-dispersed easily by simple mixing with a spatula. Its stability with regard to the molecular mass of the PLA was investigated by GPC. We found that the molecular weight decreased from 113 down to 102 kDa within eight weeks. In the same time span, the DSC properties of the latex remained stable or altered only marginally, both in the first, as well as in the second heating cycle (see Figure 2 and Table 1). These results show that degradation, if any, is only a marginal problem, and the PLA/MMT latex can be stored at room temperature. Moreover, as seen in Table 1, the degree of crystallinity of the as-prepared film is very low (~1%) and is not expected to have any influence on the mechanical or permeability properties of the films.

From Figure 2, it is clear that most of the melting enthalpy in the first heating cycle originates from the material, which has crystallized in that cycle, so that the original material presented a very low degree of crystallinity (Table 1). Moreover, one may observe a very pronounced enthalpic relaxation at *T*_g_, typical from PLA films prepared by solvent casting [8]. This relaxation vanishes in the second heating cycle. The thermal properties of the water-borne PLA/MMT films resemble those of the solvent-casted one, with the exception of the melting temperature. This is probably caused by a different crystal structure when the film is crystallized from a solution or from a latex, leading to crystallites with different melting points.

The viscosity of the latex was below 200 cP, making it suitable for different paper coating techniques. In order to obtain an appropriate viscosity, a prerequisite for a homogeneous film on the paper, the addition of the plasticizer (tributyl citrate) was mandatory.

**Figure 3:**
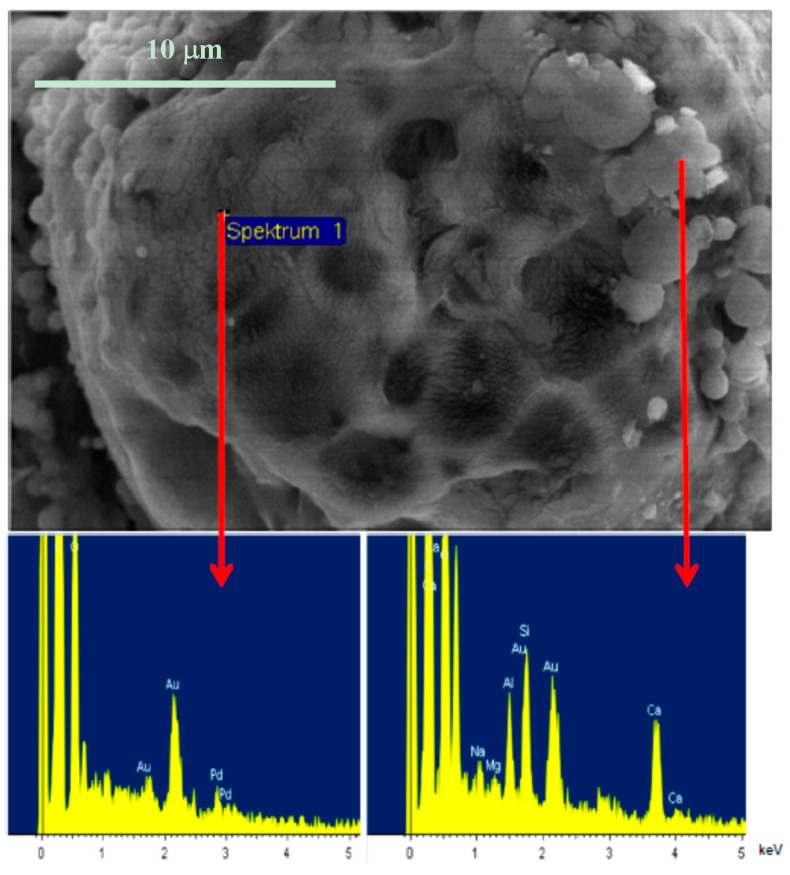
Top: SEM photo of a burst (due to high temperatures during sample preparation) PLA/MMT hybrid latex particle. **Bottom left**: EDX spectrum of the PLA region. **Bottom right**: EDX spectrum of the MMT region, which is only located at the original surface of the spherule.

**Figure 4:**
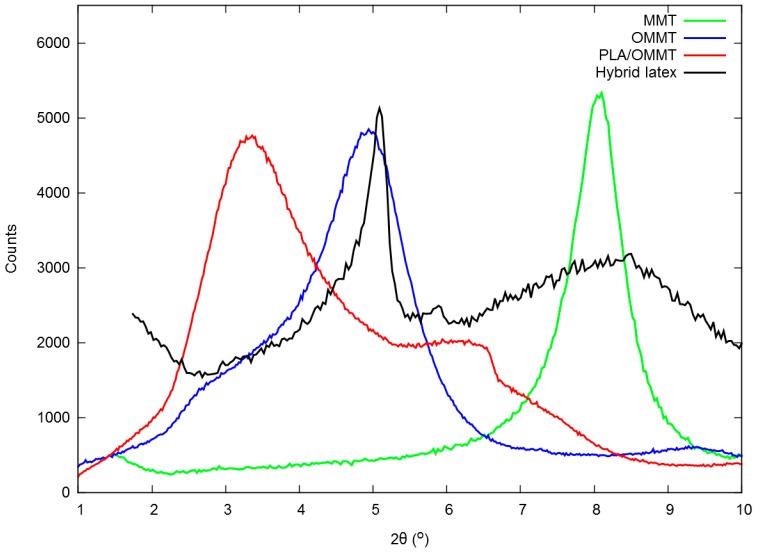
XRD spectra of clays and their mixtures with PLA. The d_001_ are 1.1 nm (MMT), 2.1 nm (organically-modified MMT (OMMT)) and 3.1 nm (PLA/OMMT). The hybrid latex presents two diffraction peaks, similar to the d_001_ of both clays (MMT and OMMT). The spectra were vertically adjusted to display similar count values.

The composition of the hybrid particles was investigated by SEM, EDX and XRD. The investigations made clear that the MMT platelets cover the surface of the spherules; they are not present in the interior. An illustration of this fact is shown in Figure 3. The SEM/EDX analysis showed that clay platelets are clearly identified on the surface of the PLA particles, as confirmed by the appearance of sodium, calcium, aluminum and silicon peaks in the EDX spectra (Figure 3, bottom right). On the other hand, no changes were observed in the XRD spectra of the hybrid film from PLA/MMT latex when compared to the pure MMT or OMMT (Figure 4). The basal spacing of the clay (defined as the d_001_ reflections in the diffractograms) was either identical to the pristine MMT (that is, 1.1 nm) or similar to the reflection of the OMMT (~2.1 nm). This data clearly suggest that the clay added was either unchanged or being intercalated only by the surfactant, as happens in the fabrication procedure of organically-modified MMT. [17] A shift to higher basal spacings (lower angles) would be expected if PLA would intercalate between the clay layers, as we have observed for hybrid films prepared from organic dispersions (d_001_ = 3.1 nm) [8]. The absence of intercalation means that PLA and MMT do not interact at the molecular/atomic level, and therefore, clay platelets are only present on the surface of the PLA particle (physical adsorption), due to the presence of surfactants. This result agrees with those found in the literature, where numerous works report that it is not possible to intercalate pristine, hydrophilic MMT with polylactide [11].

We also tried organically-modified MMTs, since these are known to interact well with PLA [8,11]. However, no latex could be prepared in this case, since the OMMT interacts very strongly with the surfactant. Once mixed with water, the OMMT absorbed all surfactant, and a polymer agglomerate was formed instead of a latex. We therefore decided to continue with the pristine MMT.

The use of hydrophilic MMT increases the stability of the latex, but it also leads to a higher (less advantageous) WVTR when the latex is coated on paper than initially desired (see Section 3.2 and Section 3.3).

### 3.2. Properties of the Neat PLA/MMT Films

The first investigations of the barrier properties were performed with neat PLA/MMT films, which had been coated on glass, dried and removed from the glass surface, as described in Section 2.3. Table 2 shows that their WVTRs decrease with decreasing content of MMT (however, PLA latexes and films without MMT were not stable, as mentioned in the Introduction). The reasons for this effect may be the brittleness of the neat films or their inhomogeneity, two properties that are supposed to increase with increasing amount of MMT. However, a WVTR of 25 g/m^2^ day was a good starting point for further tests on paper.

On the other hand, the stability of the latexes was positively influenced by the presence of MMT. As seen in Figure 3, the MMT platelets adsorbed on the surface of the PLA particles, so that less particle-particle interactions occurred. The decrease in these interactions gave a decreased tendency to agglomerate. Latexes composed of PLA and additives, but without a clay component were not stable, but tended to aggregate within a few days; the films prepared from them were not stable either. The stability of the hybrid latexes increased from a few days to several months. Although the hybrid latex tended to stratify with time, it could be re-dispersed easily by simple mixing by agitation or with a spatula.

The stability of the latexes increased with a higher amount of MMT (up to 15% of the solid content). However, their barrier properties as films decreased with increasing MMT content; see Section 3.3.

### 3.3. Properties of Papers Coated with PLA/MMT Latex

Two kinds of paper were coated with the optimized PLA/MMT formulation from Section 3.2. With six layers of PLA/MMT latex containing 3.4% MMT (see Section 2.2 and Section 2.3), coating weights of 20–22 g/m^2^ were obtained. The annealing at 150 °C improved (decreased) the WVTR; see Table 3. We assume that the thermal treatment was beneficial for the homogeneity of the coating, *i.e.*, that defects or pores, present after the coating process, disappeared.

The coatings adhered well to the paper substrates. Their WVTR properties are poorer compared to the free standing films (data in Table 2), probably due to small defects, which are still present after the annealing process and are caused due to PLA drying on the irregular paper surface, as compared to the smooth glass surface. Moreover, free-standing films were prepared in a lab-scale bar coater, while paper was coated on small industrial equipment. The latter did not allow for a fine control of film formation as did the bar-coater.

Besides improving the barrier properties of paper, the hybrid latex coatings are well suited for the quick sealing of the papers, which is an interesting feature for the food packaging industry. We performed preliminary tests that demonstrated that paper sheets coated with the latexes could be easily sealed due to the contact of the PLA layers on both paper sheets and subsequent heating, leading to a uniform film formation and, consequently, sealing. In the future, we aim to investigate the properties of multilayers of different compositions, e.g., two coatings on the paper: a pre-coating based on cellulose and the top layer made from PLA/MMT latex. In such a system, the nanofibrillated cellulose would act as an oxygen barrier layer [18], and the PLA/clay system would provide the water vapor barrier properties.

## 4. Conclusions and Outlook

The improvement of the barrier properties for food packaging by the use of biopolymer-based dispersions presented many challenges, including the strict technical requirements of the industry, such as the solid content or the viscosity of the coating latex. Nevertheless, we were able to produce a hybrid aqueous formulation with a solid content of up to 22% that could be applied on paper. This formulation contained 88.2% PLA, 5.3% tributyl citrate, 3.1% surfactants and 3.4% MMT. The PLA/MMT latex could be coated at the pilot-plant scale on two different types of paper. The coatings showed promising properties with regard to the WVTR, to adhesion and to rapid sealability. The materials used for the coating are non-toxic; thus, they are well suited for the food industry. We believe that a further optimization of the processing and drying conditions, as well as a further increase in the solid content (achieved by further optimization of the hybrid latex preparation) could yield better WVTR values.

## Figures and Tables

**Table 1:** Thermal properties of polylactides (PLA)/montmorillonite (MMT) latexes after 1 and 8 weeks of aging.

Aging Time	Molecular Weight (10^3^ g/mol)	T_g_ (°C) 1^st^/2^nd^ Heating	T_c_ (°C) 1^st^/2^nd^ Heating	T_m_ (°C) 1^st^/2^nd^ Heating	Χ_c_ (%) 1^st^ Heating
1 week	113	53/43	109/119	148/144	1.1
8 weeks	102	54/43	103/113	148/147	1.0
Solvent casted (from [8])	–	53/–	105/–	167/–	2

**Table 2:** Water vapor barrier properties of bare PLA/MMT films.

Total Solid Content of the Latex %	Amount of MMT in the Latex %	WVTR* g/(m^2^·day)	WVP ** (10^−14^ kg·m/(m^2^·Pa·s))
21	3	25	0.77
22	5	35	1.9
21	5	42	1.4
23	12	426	8.8
26	14	361	7.2

* Water vapor transmission rate. ** Water vapor permeability.

**Table 3:** WVTR of uncoated and coated papers. KL, Kraft Lux, SiNQ, Silico NQ.

Paper	Coating Weight g/m^2^	WVTR g/(m^2^ day)
KL uncoated	–	264
KL with 6 layers PLA/MMT	20	81
KL with 6 layers, annealed at 150 °C	20	42
SiNQ	–	308
SiNQ with 6 layers PLA/MMT	22	11
SiNQ with 6 layers, annealed at 150 °C	22	93
